# MetaSV: an accurate and integrative structural-variant caller for next generation sequencing

**DOI:** 10.1093/bioinformatics/btv204

**Published:** 2015-04-10

**Authors:** Marghoob Mohiyuddin, John C. Mu, Jian Li, Narges Bani Asadi, Mark B. Gerstein, Alexej Abyzov, Wing H. Wong, Hugo Y.K. Lam

**Affiliations:** ^1^Bina Technologies, Roche Sequencing, Redwood City, CA 94065, USA,; ^2^Program in Computational Biology and Bioinformatics, Yale University, New Haven, CT 06520, USA,; ^3^Department of Health Sciences Research, Center for Individualized Medicine, Mayo Clinic, Rochester, MN 55905, USA,; ^4^Department of Statistics, Stanford University, Stanford, CA 94035, USA and; ^5^Department of Health Research and Policy, Stanford University, Stanford, CA 94035, USA

## Abstract

**Summary:** Structural variations (SVs) are large genomic rearrangements that vary significantly in size, making them challenging to detect with the relatively short reads from next-generation sequencing (NGS). Different SV detection methods have been developed; however, each is limited to specific kinds of SVs with varying accuracy and resolution. Previous works have attempted to combine different methods, but they still suffer from poor accuracy particularly for insertions. We propose MetaSV, an integrated SV caller which leverages multiple orthogonal SV signals for high accuracy and resolution. MetaSV proceeds by merging SVs from multiple tools for all types of SVs. It also analyzes soft-clipped reads from alignment to detect insertions accurately since existing tools underestimate insertion SVs. Local assembly in combination with dynamic programming is used to improve breakpoint resolution. Paired-end and coverage information is used to predict SV genotypes. Using simulation and experimental data, we demonstrate the effectiveness of MetaSV across various SV types and sizes.

**Availability and implementation:** Code in Python is at http://bioinform.github.io/metasv/.

**Contact:** rd@bina.com

**Supplementary information:** Supplementary data are available at *Bioinformatics* online.

## 1 Introduction

SVs have been implicated in contributing to genomic diversity as well as genomic disorders (Stankiewicz and Lupski, 2010). Therefore, a significant amount of work has been done on detecting SVs. Generally, a tool for detecting SVs uses one or more of the following signals from read alignments: split-read [reads with split alignments, e.g. Pindel (Ye *et* *al.*, 2009)], read-pair [abnormal paired-end alignments, e.g. BreakDancer (Chen *et* *al.*, 2009)], depth-of-coverage [abnormal coverages, e.g. CNVnator (Abyzov *et* *al.*, 2011)], junction-mapping [alignments to known SV breakpoints, e.g. BreakSeq2 (Abyzov *et al.*, 2015; Lam *et al.*, 2010)] or assembly around potential breakpoints [e.g. MindTheGap (Rizk *et* *al.*, 2014)]. However, there is no signal that comprehensively detects all types of SVs since each has a niche of SV types and sizes where it works well. This necessitates the development of tools which integrate multiple methods to improve SV detection.

Prior work (Lam *et al.*, 2012; Mills *et* *al.*, 2011) has shown that variant calls made by multiple tools and methods generally are more accurate. For this reason, tools have been developed to employ multiple methods, e.g. DELLY (Rausch *et* *al.*, 2012), LUMPY (Layer *et* *al.*, 2014) and those that merge the results from multiple tools, such as SVMerge (Wong *et* *al.*, 2010). However, LUMPY and DELLY are unable to detect insertions and SVMerge ignores the SV resolution of individual tools when merging calls. Our work, therefore, attempts to address the limitations of existing SV merging tools for detecting SVs of different types and sizes with high accuracy and resolution.

## 2 Methods

MetaSV uses multiple SV-detection methods and tools to find a high-confidence and precise SV callset. The novelty of MetaSV lies in the combination of the following key ideas: calls reported by multiple orthogonal methods are generally better quality and that local assembly with dynamic programming can be used to refine the SV breakpoints.

### 2.1 Multi-method SV detection

**Fig. 1.**
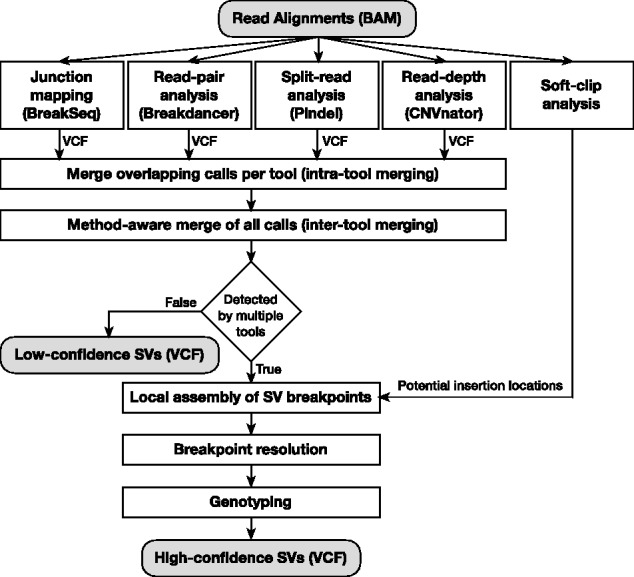
High-level view of the MetaSV methodology

MetaSV proceeds in the following steps (Fig. 1):

- **Intra-tool merging:** Potential duplicate calls generated by the same tool are merged here. Note that two calls are considered duplicate if they have significant overlap.
- **Inter-tool merging:** Calls which are generated by multiple tools are merged together. While determining the breakpoints for calls common to multiple tools, priority is given to methods known to be precise, e.g. split-read over read-pair. Note that this method-aware merging is crucial to ensuring that the breakpoints of the SVs reported are as precise as possible.
- **Local assembly:** Local assembly is performed on the SV regions reported by the tools to gather additional evidence as well as determine the SV sequences. The SPAdes (Bankevich *et* *al.*, 2012) assembler is used due to its unique ability to use paired-end information for assembly.
- **Breakpoint resolution:** The assembled SV sequences are aligned against the reference to detect or refine the breakpoints using dynamic programming (Abyzov and Gerstein, 2011).
- **Genotyping:** Read coverage around the SV breakpoints are used to determine the zygosity of the SVs. The final output is then generated as a VCF file with the genotypes for each SV.
- **Annotation:** MetaSV standardizes the inputs as well as the outputs in VCF. Each SV is annotated to indicate the corresponding calls made by the individual tools and to classify its confidence level. SVs which are detected by multiple tools, are considered high-confidence.

### 2.2 Insertion detection enhancement

The overall sensitivity of MetaSV by simply merging calls from multiple tools is upper bounded by the sensitivity of the union of all SVs detected by the individual tools. Therefore, for long insertions, which are underestimated by existing tools due to ascertainment bias, we augmented MetaSV with a soft-clip based method to boost insertion detection sensitivity. Soft-clips in read alignments are used to generate a set of candidate insertion intervals. These intervals are processed during the local assembly step to generate the final set of insertion locations. Even though assembly would not be able to determine insertion lengths for long insertions due to short read length, their locations can still be predicted accurately, which provides valuable information for interpretation. The Supplementary Text describes our method in more detail.

## 3 Results

**Fig. 2.**
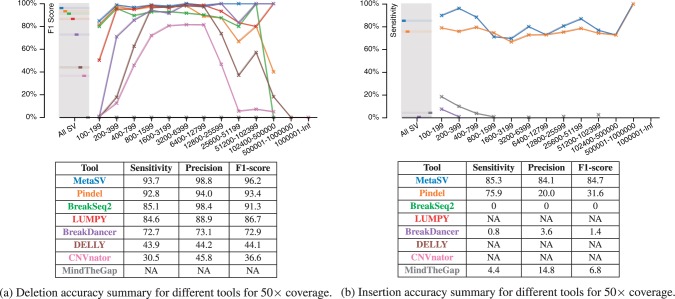
Accuracy comparisons for deletions and insertions. Accuracy metrics are shown on a per size bin basis in the plots. The tables below the plots show the aggregate accuracy scores. If a tool does not support detecting the SV type, an NA is indicated in the table. Each tool name is color coded to match the color code in the plots. DELLY’s suboptimal deletion performance was due to its lower breakpoint resolution. For insertions, although Pindel’s sensitivity was close to MetaSV, it had a significantly lower precision and overall accuracy

We demonstrate the effectiveness of MetaSV using the VarSim simulation and validation framework (Mu *et* *al.*, 2014). Simulated 2 × 100 bp NGS reads were generated at 50× coverages for the NA12878 genome using published variant sets. The reads were aligned using BWA-MEM. For comparing reported SVs against the ground truth, we use a reciprocal overlap of 90% and a wiggle of 100 bp which captures accuracy at a high breakpoint resolution. The SV size cutoff was set to 100 bp since smaller variants are a target of indel callers such as GATK’s HaplotypeCaller. Our results show that each method has varying performance in different SV size ranges. By integrating multiple methods, MetaSV achieved a steady performance across all sizes (Fig. 2). We report accuracy as F1-score, which is the harmonic mean of sensitivity and precision. For this dataset, MetaSV achieved an F1-score of 96.2% (sensitivity and precision were 93.7 and 98.8%, respectively) for deletions, indicating high accuracy and resolution. For insertions, it achieved an F1-score of 84.7% (sensitivity and precision were 85.3 and 84.1%, respectively) comparing to less than 65% for all the individual tools analyzed. Insertion length was omitted from the accuracy analysis since long insertions cannot be assembled completely with NGS reads. Nevertheless, the significantly enhanced detection of insertion events can definitely improve interpretation largely as they may cause impactful disruption in the genome. Finally, genotyping accuracies of 95.2 and 95.5% were achieved for deletions and insertions, respectively.

Figure 2 shows detailed accuracy comparisons for both deletion and insertion detection across different SV sizes and tools. For deletions (Fig. 2a), MetaSV performance was the highest across all SV sizes. In most cases, it improves upon the best performing individual tool for a given size. For insertions (Fig. 2b), the improvement due to MetaSV was more significant since all tools (with the exception of Pindel) almost detect no insertions due to inherent limitations of the methods used. Therefore, almost all improvement in accuracy is due to our insertion detection enhancement—our soft-clip based approach is very sensitive while the assembly step for insertion detection yields high precision in contrast to Pindel which had low precision. As with deletions, small insertions are more difficult to detect, in general. Detailed accuracy comparisons for other SV types are discussed in the Supplementary Text.

In order to study the impact on accuracy and runtime of varying coverages, we generated additional simulation datasets with 2 × 100 bp reads at 10× and 30× coverages. We also generated 2 × 250 bp reads at 50× coverage to investigate the impact of increasing read length for the same coverage. Although accuracy dropped for lower coverages, MetaSV was still the most stable and most accurate, with deletion F1-scores of 89.1 and 95.8% for 10× and 30× coverages, respectively. For 250 bp read length, F1-scores of 96.8 and 80.9% were achieved for deletions and insertions, respectively—insertion accuracy dropped slightly over 100 bp reads due to reduced read count for the same coverage. Furthermore, it took around 25 h to run all the four aforementioned tools for MetaSV as well as MetaSV on a single node with dual-hexcore X5675 Intel Xeon processors for 50× coverage. Because MetaSV is parallelized in all its steps, its speed should scale linearly with the number of available cores.

In addition to simulation, we used the publicly available Illumina Platinum Genomes sequencing data for NA12878 as a real testbed. Due to the lack of high-confidence comprehensive SV calls, particularly for insertions, false positive rates cannot be accurately determined using real data. Therefore, only sensitivity for deletions was reported here. For deletions, a sensitivity of 90.2% was achieved against the Complete Genomics high-confidence callset for NA12878 (Drmanac *et* *al.*, 2009) generated using their analysis pipeline version 2.0, which is similar to our simulation results. Complete Genomics was used since it is an orthogonal sequencing platform, providing a less biased validation.

## 4 Conclusions

MetaSV significantly improves the accuracy of SV-calling by integrating orthogonal methods and tools. In addition, it is augmented with soft-clip based insertion detection for significantly higher accuracy compared with the state of the art.

We consider MetaSV as a proof of concept of the effectiveness of using an ensemble approach for calling SVs. The approach is not limited to only using the four aforementioned tools—it can be easily adapted to use additional or even a different set of tools.

## Funding

W.H.W. was supported by National Institute of Health grants [1R01HG007834] and [1R01GM109836].

*Conflicts of Interest:* W.H.W. and N.B. are co-founders of Bina Technologies. W.H.W. and M.B.G. are scientific advisors for Bina Technologies.

## Supplementary Material

Supplementary Data
