# Supplementary material for: MetaSV: an accurate and integrative structural-variant caller for next generation sequencing
Source: Bioinformatics. 2015 Apr 10;31(16):2741–4. doi: 10.1093/bioinformatics/btv204 (PMC4528635; doi:10.1093/bioinformatics/btv204)
Supplement: Supplementary Data [file supp_31_16_2741__index.html]

MetaSV: An accurate and integrative structural-variant caller for next generation sequencing — MetaSV: an accurate and integrative structural-variant caller for next generation sequencing — MetaSV: an accurate and integrative structural-variant caller for next generation sequencing — Supplementary Data 

# MetaSV: an accurate and integrative structural-variant caller for next generation sequencing

## Supplementary Data

files

**Files in this Data Supplement:**

- Supplementary Data - pdf file
