# Supplementary material for: MetaSV: an accurate and integrative structural-variant caller for next generation sequencing
Source: Bioinformatics. 2015 Apr 10;31(16):2741–4. doi: 10.1093/bioinformatics/btv204 (PMC4528635; doi:10.1093/bioinformatics/btv204)
Supplement: Supplementary Data [file supp_btv204_metasv_supp.pdf]

MetaSV: An accurate integrative structural-variant caller for next generation sequencing

SUPPLEMENTARY INFORMATION

Marghoob Mohiyuddin*, John C. Mu*, Jian Li,
Narges Bani Asadi, Mark B. Gerstein, Alexej Abyzov,
Wing H. Wong, and Hugo Y.K. Lam†

1 Insertion Detection Enhancement

Given the poor performance of existing tools in detecting insertions, we augmented MetaSV with an option to enhance insertion detection using soft-clips in read alignments. Large soft-clips in reads are considered evidence of long insertions. In fact, they can also be evidence of other SVs, e.g., duplications, inversions or translocations, but we focus on insertions in this work. Further validation is done by performing local assembly around the potential insertion locations followed by dynamic programming to resolve the insertion location precisely. Figure 1 shows the workflow of the our insertion detection method. The steps involved are discussed in more detail below:

1. Process the read alignments looking for large soft-clips in reads mapped with good quality. The location of the soft-clip is considered a candidate insertion location. In addition, the soft-clipped bases must be of high quality to reduce false hits due to sequencing errors. At the end of this step, each candidate read generates an interval for assembly centered at the soft-clip location.

2. Overlapping intervals in Step 1 are merged which drastically reduces the number of intervals to process–the number of intervals in Step 1 used to generate a merged interval is considered the support count of the merged interval. To further reduce false hits and computational cost, merged intervals with low support count are discarded.

3. Local assembly is perfomed on the insertion intervals from Step 2. This is done by extracting read pairs with at least one end mapped in and around the intervals of interest. Note that assembly will generate potentially multiple contigs for the same intervals. In case of a heterozygous insertion, it is possible that assembly may fail if the reads supporting the insertion allele are few in comparison to the reference allele, especially for large insertions. Therefore, assembly is performed twice with different sets of reads for the insertion interval: once with all the reads extracted from the interval and a second time with only the imperfectly mapped reads in order to improve the sensitivity towards heterozygous insertions.

4. Dynamic programming (Abyzov and Gerstein, 2011) is used to precisely determine the insertion locations by aligning the assembled contigs from Step 3 against the reference. A contig is considered good if it aligns with a large insertion close to the predicted insertion locations from Step 1 (Figure 2a). Note that if no good contig can be found for an insertion interval, then the interval is considered a false positive and discarded. In order to decrease false positives, it is also required for the assembled contigs of an insertion interval to be consistent with each other, i.e., they must indicate almost the

*The authors wish it to be known that, in their opinion, the first two authors should be regarded as joint First Authors.
†To whom correspondence should be addressed.

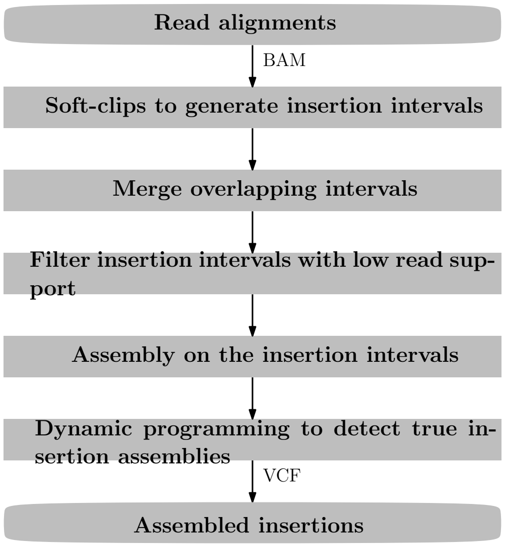

Figure 1: High-level view of insertion detection.

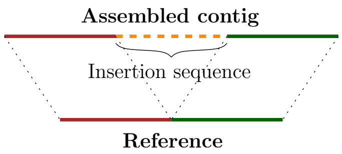

(a) An assembly supporting a small insertion. The assembled contig must align against the reference around the insertion location with an insertion.

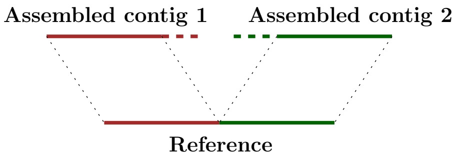

(b) A pair of assemblies supporting a large insertion. The assembled contigs must align on different sides of the insertion location. In addition, they must have a significant portion unaligned in order of avoid assemblies which are exactly the reference.

Figure 2: Different kinds of assemblies for short and long insertions.

same insertion location. For long insertions, it is difficult to assemble the whole insertion sequence. For these cases, pairs of contig alignments are considered (Figure 2b)–if there are long fragments from two different assembled contigs which align close to a potential insertion location and the fragments align on opposite sides of the location, then there is evidence of a long insertion from local assembly. In summary, an insertion is called at a location if the assembled contigs either align against the reference with a long insertion or pairs of contigs can be found which align with long fragments on different sides of the location.

We note that Steps 3 and 4 are done together for both insertions and other SV types.

2 Simulation Results

We use the VarSim framework (Mu *et al.*, 2014) to simulate the NGS reads for comparing the various SV detection tools. Simulated 2×100bp paired-end NGS reads were generated at 50× coverage with the ART simulator using the read base error profiles from the Illumina Platinum Genomes NA12878 sample. Insert size mean and standard deviation were 350bp and 50bp respectively. The ground truth was constructed as

follows:

- Small variants (SNPs and small indels) were obtained from the Genome in a Bottle Consortium high-confidence calls for NA12878 (Zook *et al.*, 2014).
- Deletion SVs were obtained from the 1000Genomes project data (Abecasis *et al.*, 2010; Mills *et al.*, 2011).
- Insertion SVs were generated by randomly sampling the locations from DGV (MacDonald *et al.*, 2014) and the sequences from the concatenation of the Venter insertion sequences (Levy *et al.*, 2007).
- Other SVs were randomly sampled from DGV.

The simulated FASTQs were aligned using BWA-MEM (Li, 2013) (version 0.7.12-r1039) and then processed by the various SV-calling tools, including MetaSV. For MetaSV local assembly SPAdes (Bankevich *et al.*, 2012) version 3.5.0 was used. The dynamic programming step in MetaSV for assembled contigs was performed using a modified AGE (Abyzov and Gerstein, 2011) at `https://github.com/marghoob/AGE/tree/simple-parseable-output`–AGE was modified to make the output easier to parse for MetaSV.

We compare MetaSV against the state of the art in SV detection. The following tools were included in the comparison: BreakDancer (Chen *et al.*, 2009), BreakSeq2 (Abyzov *et al.*, 2015), Pindel (Ye *et al.*, 2009), CNVnator (Abyzov *et al.*, 2011), LUMPY (Layer *et al.*, 2014), DELLY (Rausch *et al.*, 2012) and MindTheGap (Rizk *et al.*, 2014). Table 1 provides a summary of the tools and the versions used–default settings were used when running the individual tools. In this work, the outputs of BreakDancer, BreakSeq2, CNVnator and Pindel were provided as inputs to MetaSV to generate accurate SV calls. Insertion detection enhancement was also turned on for the accuracy comparison. Tables 2, 3, 4 and 5 show the accuracies for the individual tools. We can clearly see that MetaSV achieves significantly higher accuracy in comparison to other tools for deletions, insertions and tandem duplications. For inversions, however, the accuracy is lower since the consensus accuracy is limited by BreakDancer. Future work will leverage the soft-clip based approach in Section 1 to improve the accuracy of inversion detection.

2.1 Impact of Coverage

In addition to the primary results on 50× coverage simulated data, simulation was also performed on 10× and 30× coverages to investigate the impact on SV detection accuracy as coverage is varied. Furthermore, 250bp paired-end simulated reads at 50× coverage was also done to study the impact of increased read-length on SV detection accuracy. Figures 3 and 4 show how the accuracy (F1-score) of deletion and insertion detection varies for each tool as coverage is varied from 10× to 50×. As expected, most of the tools, including MetaSV, improve in accuracy as coverage increases due to increased read support for SV detection. We also note that MetaSV still has the best performance across all coverages for both insertions and deletions–it also achieves the most stable performance when coverage is varied. For insertions, MetaSV's improvement over other tools is more significant.

2.2 Impact of Read Length

Figures 5 and 6 show the accuracies for 250bp paired-end simulation at 50× coverage–MetaSV achieves F1-scores of 96.8% and 80.9% for deletion and insertion detection respectively. We also note that MetaSV performance improvement over other tools in this case is better than the 100bp simulation at 50× coverage. Since the coverage was kept constant, the number of reads decreased by a factor of 2.5× which means reduced sensitivity for other SV-calling tools. MetaSV, however, is able to maintain accuracy since it integrates across four SV-caling signals which means increased tolerance to coverage and read-length variations.

2.3 Speed of MetaSV

Figure 7 shows how the time taken for MetaSV varies as coverage is varied as a stacked bar chart with the time taken to run the four individual SV-calling tools as well as the time to run MetaSV with assembly. For this performance data, benchmarking was performed on an Intel Xeon X5675 dual-hexcore machine with

**Tool**	**Version**	**Command-line options**	Breakpoint resolution
BreakDancer	1.4.5 (commit 251f983)	`-s 7 -c 3 -m 1000000000 -q 35 -r 2 -x 1000`<br>`-b 100 -y 30`	> 1bp
BreakSeq2	2.0	`--min_span 10 --window 100 --min_overlap 10`<br>`--junction_length 200`	1bp
CNVnator	0.3.1	Bin size of 100bp was used<br>*Tree generation*: -unique<br>*Histogram generation*: -his 100<br>*Stat generation*: -stat 100<br>*Partition*: -partition 100<br>*Calling*: -call 100	100bp (bin size)
DELLY	0.6.1	`-q 0 -s 9 -m 13 -u 20`	$\geq$ 1bp
LUMPY	0.2.9	`-mw 4 -tt 0.0`<br>`-pe mean:350,stdev:50,read_length:100,`<br>`min_non_overlap:100,discordant_z:4,back_distance:20,`<br>`weight:1,id:1,min_mapping_threshold:20`<br>`-sr back_distance:20,weight:1,id:2,min_mapping_threshold:20`	$\geq$ 1bp
MindTheGap	0.6447	`-k 27 -t 3 -mrep 5 -i 10000 -n 100 -m 0 -r 0 -bfs -h 1`	1bp
Pindel	0.2.5a8	`-R 1 -H 8 -T 4 -x 4 -w 5000000 -e 0.01 -E 0.95`<br>`-u 0.02 -n 2 -r 1 -t 1 -l 1 -a 1 -m 3 -v 50`<br>`-d 30 -B 0 -A 0 -M 3 -q 0 -I 0`	1bp
MetaSV	0.2-alpha	`--filter_gaps --keep_standard_contigs --wiggle 100`<br>`--inswiggle 100 --minsvlen 50 --overlap_ratio 0.5`<br>`--boost_ins  --min_ins_support 2`<br>`--min_ins_support_frac 0 --max_ins_intervals 50000`<br>`--num_threads 15`	1bp

Table 1: Tools run, versions used and their breakpoint resolution. Note that CNVnator SV-calling involves multiple invocations of the executable. For the tools mentioned, the command-line options stated are generally the default options for that version of the tool. Interchromosomal SV detection was disable to reduce run time. BreakSeq2 was run with the latest breakpoint library available from `http://sv.gersteinlab.org/phase1bkpts/`. LUMPY and DELLY parameters were tuned for best performance. Breakpoint resolution varies across the tools. Since DELLY and LUMPY use a combination of SV signals, their breakpoint resolution can vary depending on the signals used for detecting an SV. The human reference genome build 37 with the decoy contig was used for alignment as well all SV-calling. As much as possible, only the major contigs chr1, chr2, . . ., chr22, chrX, chrY and chrMT were processed for minimum processing time.

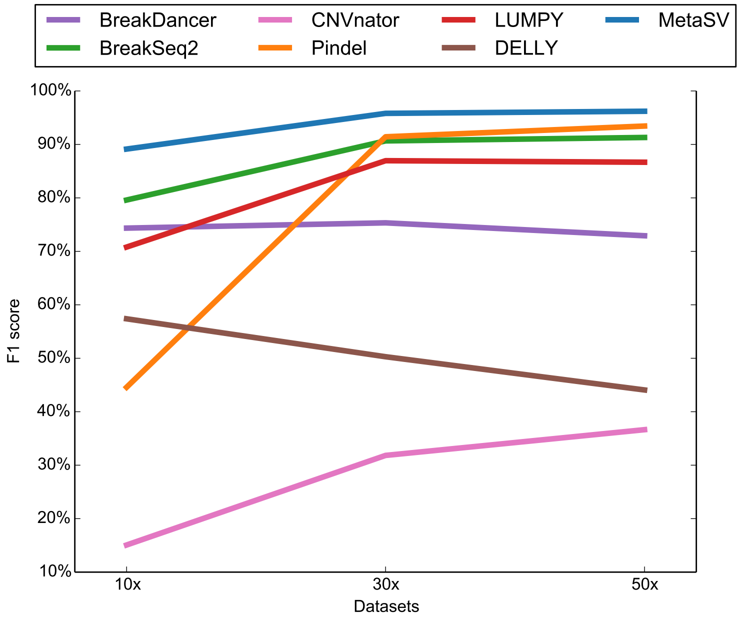

Figure 3: Deletion detection accuracy for different coverages.

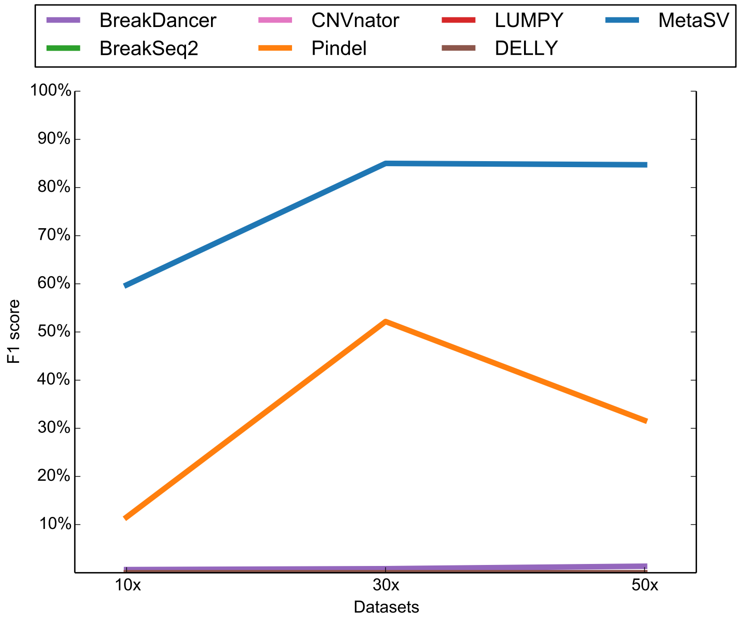

Figure 4: Insertion detection accuracy for different coverages. Note that Pindel achieves best accuracy at 30× coverage which appears anomalous–this is due to the improved FDR at 30× coverage over 50× coverage.

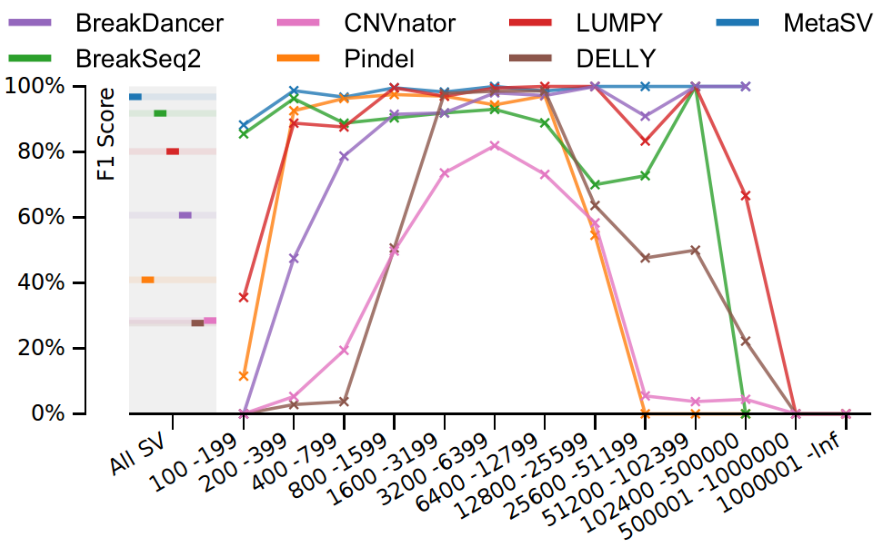

Figure 5: Deletion detection accuracy for 250bp paired-end simulation at 50× coverage.

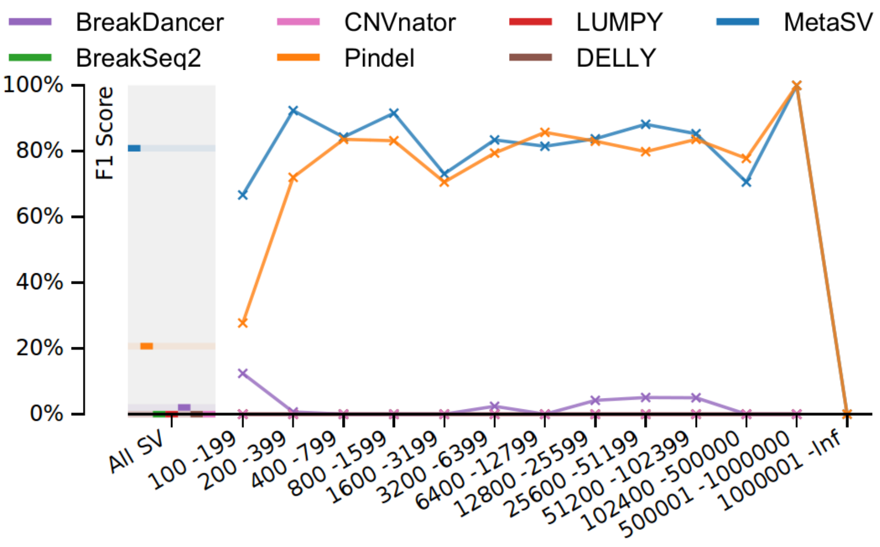

Figure 6: Insertion detection accuracy for 250bp paired-end simulation at 50× coverage. Note that Pindel has a significantly low precision due to a large number false large insertions.

Tool	Reported	True positives	False positives	Sensitivity	Precision	F1-score
**MetaSV**	1192	1178	14	93.7	98.8	96.2
**Pindel**	1353	1161	92	92.4	92.7	92.5
**BreakSeq2**	1102	1078	24	85.8	97.8	91.4
**LUMPY**	1196	1063	133	84.6	88.9	86.7
**BreakDancer**	1250	914	336	72.7	73.1	72.9
**DELLY**	1248	552	696	43.9	44.2	44.1
**CNVnator**	839	384	455	30.5	45.8	36.6
**MindTheGap**	NA	NA	NA	NA	NA	NA

Table 2: Deletion detection accuracy for different tools. Total number of true deletions was 1257. Rows are sorted in order of decreasing F1-scores. DELLY's F1-score is low due to low SV resolution of the calls made. With 50% reciprocal overlap, DELLY was able to get 77.8% sensitivity, 78.5% precision and 78.1% F1-score.

Tool	Reported	True positives	False positives	Sensitivity	Precision	F1-score
**MetaSV**	1454	1223	231	85.3	84.1	84.7
**Pindel**	5437	1087	4350	75.6	20.0	31.6
**MindTheGap**	427	63	364	4.4	14.8	8.1
**BreakDancer**	334	12	322	0.8	3.6	1.4
**BreakSeq2**	0	0	0	0	0	0
**CNVnator**	NA	NA	NA	NA	NA	NA
**LUMPY**	NA	NA	NA	NA	NA	NA
**DELLY**	NA	NA	NA	NA	NA	NA

Table 3: Insertion detection accuracy for different tools. Total number of true insertions was 1433. Rows are sorted in order of decreasing F1-scores.

12 physical cores in total and 96 GB of DRAM. All the SV-calling tools were run on a per-chromosome basis–for maximum throughput, 15 processes were run at a time. Note that the limit of 15 processes was imposed due to the DRAM memory constraints. This means that the peak memory utilization was close to 96 GB. As expected, the total time increased with increase in coverage. However, the speed scaling was less than linear with coverage. We attribute this to the evidence for calling SVs scales less than linearly with increased coverage once coverage is high enough.

3 Results on Other Genomes

In order to do further validation of MetaSV, we also considered looking into other genomes, particularly the mouse dataset in the SMASH work (Talwalkar *et al.*, 2014) but the quality of the dataset limits SV detection for small SV. The mean insert size was 174bp and the standard deviation was 134bp after aligning with BWA-MEM which would limit small SV detection given the large standard deviation. Since the small SVs dominate, the F1-scores for all the tools would be poor. In addition, we also encountered problems in running the tools on the mouse genome reference, e.g., Pindel incurred a segmentation fault. Although our approach is not limited to only human genomes, most of the popular SV detection tools have been tested mostly on the human genome. This means the effectiveness of our approach is best demonstrated on the human genome. Due to lack of good support for other genomes, we omit non-human genomes from our comparisons.

Tool	Reported	True positives	False positives	Sensitivity	Precision	F1-score
**LUMPY**	60	59	1	70.2	98.3	81.9
**Pindel**	86	69	17	82.1	80.2	81.2
**MetaSV**	49	46	3	54.8	93.9	69.2
**BreakDancer**	71	45	26	53.6	63.3	58.1
**DELLY**	459	83	376	98.8	18.1	30.6
**BreakSeq2**	NA	NA	NA	NA	NA	NA
**CNVnator**	NA	NA	NA	NA	NA	NA
**MindTheGap**	NA	NA	NA	NA	NA	NA

Table 4: Inversion detection accuracy for different tools. Total number of true inversions was 84. Rows are sorted in order of decreasing F1-scores.

Tool	Reported	True positives	False positives	Sensitivity	Precision	F1-score
**MetaSV**	42	38	4	84.4	90.5	87.3
**Pindel**	110	43	67	95.6	39.1	55.5
**LUMPY**	173	34	139	75.6	19.7	31.2
**DELLY**	402	40	362	88.9	10.0	17.9
**CNVnator**	416	36	380	80.0	8.7	15.6
**BreakDancer**	NA	NA	NA	NA	NA	NA
**BreakSeq2**	NA	NA	NA	NA	NA	NA
**MindTheGap**	NA	NA	NA	NA	NA	NA

Table 5: Tandem duplication detection accuracy for different tools. Total number of true tandem duplications was 45. Rows are sorted in order of decreasing F1-scores.

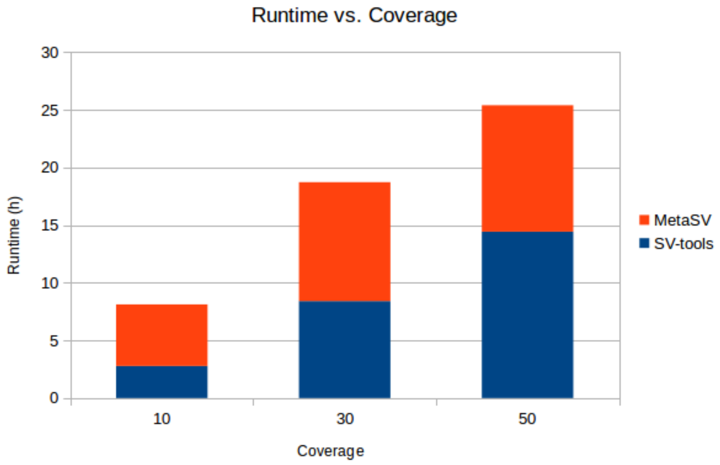

Figure 7: MetaSV and SV-calling time on a single node as coverage is varied. Note that SV tools were run on a per-chromosome basis and at a time, a maximum of 15 processes were run to maximize SV-caller throughput. For comparison, MindTheGap, which uses assembly to detect insertions, took 48 hours to run on the same node for 50× coverage. In contrast, MetaSV assembly took around 11 hours for 50× coverage.

References

Abecasis, G.R. et al (2010). A map of human genome variation from population-scale sequencing. *Nature*, **467**(7319), 1061–1073.

Abyzov, A. and Gerstein, M. (2011). AGE: defining breakpoints of genomic structural variants at single-nucleotide resolution, through optimal alignments with gap excision. *Bioinformatics*, **27**(5), 595–603.

Abyzov, A., Urban, A.E., Snyder, M. and Gerstein, M. (2011). CNVnator: an approach to discover, genotype, and characterize typical and atypical CNVs from family and population genome sequencing. *Genome research*, **21**(6), 974–984.

Abyzov, A. et al (2015). Analysis of deletion breakpoints from 1,092 humans reveals details of mutation mechanisms. *Nature Communications*. In press.

Bankevich, A. et al (2012). SPAdes: A new genome assembly algorithm and its applications to single-cell sequencing. *Journal of Computational Biology*, **19**(5), 455–477.

Chen, K. et al (2009). BreakDancer: an algorithm for high-resolution mapping of genomic structural variation. *Nature methods*, **6**(9), 677–681.

Layer, R.M., Chiang, C., Quinlan, A.R. and Hall, I.M. (2014). LUMPY: a probabilistic framework for structural variant discovery. *Genome Biol*, **15**(6), R84.

Levy, S. et al (2007). The diploid genome sequence of an individual human. *PLoS Biol.*, **5**(10), e254.

Li, H. (2013). Aligning sequence reads, clone sequences and assembly contigs with bwa-mem.

MacDonald, J.R., Ziman, R., Yuen, R.K.C., Feuk, L. and Scherer, S.W. (2014). The Database of Genomic Variants: a curated collection of structural variation in the human genome. *Nucleic Acids Research*, **42**(D1), D986–D992.

Mills, R.E. et al (2011). Mapping copy number variation by population-scale genome sequencing. *Nature*, **470**(7332), 59–65.

Mu, J.C. et al (2014). VarSim: A high-fidelity simulation and validation framework for high-throughput genome sequencing with cancer applications. *Bioinformatics*.

Rausch, T. et al (2012). DELLY: structural variant discovery by integrated paired-end and split-read analysis. *Bioinformatics*, **28**(18), i333–i339.

Rizk, G., Gouin, A., Chikhi, R. and Lemaitre, C. (2014). MindTheGap : integrated detection and assembly of short and long insertions. *Bioinformatics*, pages 1–7.

Talwalkar, A. et al (2014). SMASH: a benchmarking toolkit for human genome variant calling. *Bioinformatics*, **30**(19), 2787–2795.

Ye, K., Schulz, M.H., Long, Q., Apweiler, R. and Ning, Z. (2009). Pindel: a pattern growth approach to detect break points of large deletions and medium sized insertions from paired-end short reads. *Bioinformatics*, **25**(21), 2865–2871.

Zook, J.M. et al (2014). Integrating human sequence data sets provides a resource of benchmark SNP and indel genotype calls. *Nat Biotechnol*, **32**(3), 246–251.
